# VDAC2 malonylation participates in sepsis-induced myocardial dysfunction via mitochondrial-related ferroptosis

**DOI:** 10.7150/ijbs.84613

**Published:** 2023-06-14

**Authors:** Han She, Lei Tan, Yuanlin Du, Yuanqun Zhou, Ningke Guo, Jun Zhang, Yunxia Du, Yi Wang, Zhengbin Wu, Chunhua Ma, Qinghui Li, Qingxiang Mao, Yi Hu, Liangming Liu, Tao Li

**Affiliations:** 1 State Key Laboratory of Trauma, Burns and Combined Injury, Shock and Transfusion Department, Daping Hospital, Army Medical University, Chongqing400042, China; 2 Department of Anesthesiology, Daping Hospital, Army Medical University, Chongqing400042, China; 3 Department of Intensive care unit, Daping Hospital, Army Medical University, Chongqing400042, China

**Keywords:** Sepsis, Malonylation, VDAC2, Ferroptosis, Malonyl-CoA

## Abstract

Sepsis-induced myocardial dysfunction (SIMD) is a prevalent and severe form of organ dysfunction with elusive underlying mechanisms and limited treatment options. In this study, the cecal ligation and puncture and lipopolysaccharide (LPS) were used to reproduce sepsis model *in vitro* and vivo. The level of voltage-dependent anion channel 2 (VDAC2) malonylation and myocardial malonyl-CoA were detected by mass spectrometry and LC-MS-based metabolomics. Role of VDAC2 malonylation on cardiomyocytes ferroptosis and treatment effect of mitochondrial targeting nano material TPP-AAV were observed. The results showed that VDAC2 lysine malonylation was significantly elevated after sepsis. In addition, the regulation of VDAC2 lysine 46 (K46) malonylation by K46E and K46Q mutation affected mitochondrial-related ferroptosis and myocardial injury. The molecular dynamic simulation and circular dichroism further demonstrated that VDAC2 malonylation altered the N-terminus structure of the VDAC2 channel, causing mitochondrial dysfunction, increasing mitochondrial ROS levels, and leading to ferroptosis. Malonyl-CoA was identified as the primary inducer of VDAC2 malonylation. Furthermore, the inhibition of malonyl-CoA using ND-630 or ACC2 knock-down significantly reduced the malonylation of VDAC2, decreased the occurrence of ferroptosis in cardiomyocytes, and alleviated SIMD. The study also found that the inhibition of VDAC2 malonylation by synthesizing mitochondria targeting nano material TPP-AAV could further alleviate ferroptosis and myocardial dysfunction following sepsis. In summary, our findings indicated that VDAC2 malonylation plays a crucial role in SIMD and that targeting VDAC2 malonylation could be a potential treatment strategy for SIMD.

## Introduction

Sepsis is a severe life-threatening condition where organ function is disrupted due to a dysregulated host response to infection^1^. It is a daunting challenge to human health, affecting approximately 48.9 million patients and causing 11 million deaths annually^2^-^3^. Sepsis-induced myocardial dysfunction (SIMD) is a serious complication of sepsis, leading to poor prognosis^4^-^5^. The mortality rate for SIMD patients is considerably higher than those without the complication (70% vs. 20%). Mainly, SIMD leads to a decline in myocardial contractility, inadequate organ perfusion, and multiple organ failure. Inflammatory responses, Ca^2+^ dysregulation, mitochondrial dysfunction, autonomic dysfunction, excessive production of nitric oxide (NO), and apoptosis are recognized mechanisms of SIMD^7^-^11^. While conventional therapies like norepinephrine, calcium sensitizer, and beta-blockers have achieved some progress in treating SIMD, their therapeutic effects remain less than ideal^12^-^14^. Therefore, elucidating SIMD's mechanism, identifying effective therapeutic targets and searching for targeted therapy strategies is of great significance.

Post-translational modification (PTM) of protein lysine residues, including succinylation, acetylation, and crotonylation, plays a critical role in regulating cellular functions in both physiological and pathological states^15^. Lysine malonylation (Kmal) is a novel and conservative lysine PTM widely found in eukaryotic and prokaryotic cells. Initially identified in HeLa cells and Escherichia coli using the anti-malonyllysine antibody^16^, the advancement of detection technologies has revealed Kmal's critical roles in inflammation, Type 2 diabetes, and angiogenesis^17^-^18^. A recent study also linked Kmal to cardiac hypertrophy^19^. However, the potential involvement of Kmal during sepsis-induced myocardial dysfunction is yet to be explored.

Ferroptosis is a type of cell death triggered by ferrous ions (Fe^2+^) due to the accumulation of reactive oxygen species and lipid peroxides^20^. Recent studies establish that ferroptosis is a crucial target in treating heart diseases. Fang et al. discovered that ferristatin-1 (Fer-1), a specific ferroptosis inhibitor, significantly reduced doxorubicin-induced cardiac toxicity, ultimately improving mouse survival rates^21^. In a separate study, Gao et al. demonstrated that by down-regulating glutamine metabolism to inhibit ferroptosis, the injury to the heart can be reduced in their isolated mouse cardiac ischemia-reperfusion model^22^. Consequently, targeting ferroptosis is a vital approach to protect cardiac function, with considerable research and translational significance.

Mitochondria are essential organelles in cells and closely linked to ferroptosis in recent studies. Treatment with doxorubicin causes significant changes in mice's mitochondrial morphology and function, but Fer-1 can restore these changes^21^. Additionally, myocardial mitochondria separate the accumulation of iron and lipid peroxidation, which primarily occurs in them^21^. Although one study has linked sepsis-induced myocardial injury to ferroptosis, the regulation mechanism is unclear^23^.

The study aims to explore a new regulatory target for treating SIMD by exploring the relationship between protein lysine malonylation and mitochondrial-related ferroptosis. We will also investigate the potential therapy strategy targeting the mechanism.

## Materials and Methods

### Ethics statement

The relevant human study was approved by the Ethics Committee of the Research Institute of Surgery and was registered with the Chinese Clinical Trial Registry (ChiCTR2200055772). Animal experiments were conducted following the guidelines of Animal Research: Reporting of *In vivo* Experiments (ARRIVE), and were approved by the Laboratory Animal Welfare and Ethics Committee of Army Medical University (Approval No. AMUWEC20224867).

### Reagents

Antibody for VDAC2 (GTX104745) was purchased from GeneTEX (San Antonio, TX, USA). Antibodies for Malonyl-lysine (PTM-901) and Malonyl-lysine VDAC2 K46mal (CL062201) were obtained from PTM Biolabs (Hangzhou, China). Antibodies for CPT1 (ab128568), GPX4 (ab125066), and GAPDH (ab8245) were purchased from Abcam (Cambridge, MA, USA). Antibodies for Acetyl-CoA Carboxylase (3662S), phospho-Acetyl-CoA Carboxylase (Ser79) (3661), COX2 (12282), and Sirt5 (8782S) were purchased from Cell Signaling Technology (Danvers, Massachusetts, USA). Lipopolysaccharide (L4130), Triphenyl phosphine (T84409), 6-bromohexanoic acid (150452), N, N-dicyclohexylcarbodiimide (8.02954) and N-hydroxy succinimide (130672) were purchased from Sigma (St. Louis, MO, USA). JC-1 (C2003S), MDA (S0131M), and Glutathione detection kit (S0053) were purchased from Beyotime Biotechnology (Shanghai, China). Ferrous iron colorimetric detection kit (E-BC-K773-M) was purchased from Elab-science (Wuhan, China). VDAC2 protein (NBP1-72499) was purchased from Novus (Briarwood Avenue, Centennial, USA). ND-630 (HY- 16901) was purchased from MedChemExpress (Monmouth, NJ, America). Mito-SOX Red (M36008) was purchased from Invitrogen (Carlsbad, CA, USA). MitoBright-Deep Red (MT12) and Mito-FerroGreen (M489) were purchased from Dojindo (Kyushu, Japan). Adenoviral vector for ACC2 deletion (Ad-shACC2), Sirt5 overexpression (Ad-Sirt5 OE) and adeno-associated viruses (AAVs) were generated by Obio life Technology (Shanghai, China). Primers for ACC2 KO genotype were Forward: 5′-CcggCCGGATCACTATCGGCAATAACTCGAG TTATTGCCGATAGTGATCCGGTTTTTTg-3′ and Reverse: 5′-aattcaaaaaaCCGGATCACTATCGGCAATAACTCGAGTTATTGCCGATAGTGATCCGG-3′. All other chemicals were purchased from Sigma unless specifically mentioned.

### Sepsis patients and healthy controls recruitment

A total of 30 sepsis patients were recruited from the Department of Intensive Care Unit at Daping Hospital, based on Sepsis-3 criteria reference for sepsis and septic shock^1^. Patients were excluded from the study if they met any of the following criteria: 1) Age over 80 or under 18 years old; 2) Pregnancy or lactation; 3) Hyperlipidemia, diabetes mellitus, or other metabolic diseases; 4) Any other serious cardiovascular diseases; 5) Any other psychiatric diseases, or had a low degree of cooperation during treatment; 6) Were dependent on alcohol or drugs; 7) Had a tumor or immune deficiency and other diseases that had a greater impact on immunity; or 8) The patient or their family refused to participate in the study. Fifteen age-matched healthy volunteers were enrolled in the study from the State Key Laboratory of Trauma, Burns, and Combined Injury. All participants were admitted to the hospital between December 2021 and April 2022, and written informed consent was obtained prior to inclusion in the study. Blood samples were collected from sepsis patients within 24 hours of admission, and from healthy controls upon enrollment.

### Animal management and sepsis model establishment

Adult Sprague Dawley (SD) rats weighing 200-220g were bred in an animal facility with filtered positive-pressure ventilation and provided with ad libitum food and water. The room maintained a temperature of 23-25°C and a relative humidity of 40%-70%. The rat sepsis model was established through cecal ligation and puncture (CLP) according to a previous study^24^-^25^. After 12 hours of CLP, the rats were sacrificed, and their hearts were collected for further research.

### Cell culture and treatment

The H9C2 cardiomyocyte cell line was acquired from the American Type Culture Collection (ATCC, Manassas, VA, USA)^24^. The cells were cultured at 37°C in a humidified incubator, with 95% air and 5% CO2. DMEM (Invitrogen, CA, USA) supplemented with 10% fetal bovine serum (v/v) (Gibco, MD, USA), 100 U/ml penicillin, and 100 μg/ml streptomycin (Invitrogen, CA, USA) were utilized to maintain the cells. To establish an *in vitro* sepsis model, H9C2 cells were stimulated with 500 ng/ml LPS for 12 hours. H9C2 cells in the normal group were incubated with an equal amount of DMEM.

### Malonylation mass spectrometry

The hearts were ground with liquid nitrogen into a cell powder and transferred to a 5-ml centrifuge tube. Then, four volumes of lysis buffer were added to the cell powder, followed by three rounds of sonication on ice using a high-intensity ultrasonic processor (Scientz). The debris was removed by centrifugation at 12,000 g for 10 minutes at 4 °C, and then the supernatant was collected. The protein level was quantified using a BCA kit, and 2 mg of protein from each sample was collected by depositing with 20% trichloroacetic acid (TCA) for 2 hours at 4℃. The deposit was washed with cold acetone twice and then digested with trypsin (Promega, Madison, WI) overnight at a 1:50 enzyme-to-protein ratio after adding 200 mM TEAB. The samples were reduced and alkylated with dithiothreitol (DTT) and iodoacetamide (IAA). Tryptic peptides were dissolved in IP buffer (100 mM NaCl, 1 mM EDTA, 50 mM Tris-HCl, 0.5% NP-40, PH 8.0), incubated in malonylated resin at 4℃ overnight, and then washed with IP buffer and water. The enriched malonylated peptides were eluted with 0.1% TFA, desalted using C18 Zip Tips, and then dried for MS analysis. The enriched malonylated peptides were dissolved in buffer A (0.1% (v/v) formic acid and 2% acetonitrile) and loaded onto an LC column for separation using a NanoElute UHPLC system (Bruker Daltonics) with a 60-minute gradient at 300 nl/min. Buffer B contained 0.1% (v/v) formic acid in acetonitrile. Enriched proteomic samples were analyzed on a timsTOF Pro mass spectrometer (Bruker Daltonics), equipped with a ReproSil-Pur Basic C18 column (1.9 μm, 25 cm length, 100 μm i. d.). Peptide precursor ions and secondary fragments were detected by TOF. The scan range was set at 100-1700 m/z.

### Lentiviral construction and cell transfections

Lentiviral packaging system was utilized for overexpression of VDAC2 (K46E), VDAC2 (K46Q), and VDAC2 (K46R). The vector pSLenti-SFH-P2A-Puro-CMV-Vdac2 (K46E)-3xFLAG-WPRE, pSLenti-SFH-P2A-Puro-CMV-Vdac2 (K46Q)-3xFLAG-WPRE, and pSLenti-SFH-P2A-Puro-CMV-Vdac2 (K46R)-3xFLAG-WPRE (Obio Technology, Shanghai, China) were transfected into H9C2 cell lines in accordance with the manufacturer's instructions to establish cell lines with over-expressed exogenous VDAC2. Transduced cells were selected using puromycin (EZ2811D376, BioFroxx, Germany) at a concentration of 2 μg/ml and maintained in medium with 1 μg/ml puromycin.

### Targeted acyl-CoA profiling

The frozen heart samples from rats were spiked with 10-fold volume (μl/mg) of 80% methanol in a frozen state using Tissue Lyser (JX-24, Jingxin, Shanghai, China) with beads at 40 Hz for 4 minutes. The resulting mixture was centrifuged at 15000g at 4°C for 15 minutes, and the supernatant was mixed with 50 μl of internal standard solution (acetyl-13C2-CoA, 1 μg/ml) and dried under nitrogen. The dried extract was then redissolved in 1 mL of methanol/isopropanol/acetic acid/water (9:3:4:4) and purified further by solid phase extraction. The eluates were evaporated to dryness and reconstituted in 50 μl of 50% acetonitrile (V, μl). Quality control (QC) samples were obtained by pooling all the prepared samples isometrically. Raw data were processed using the Mass Hunter Workstation Software (Agilent) by default parameters and manual inspection. The concentrations of coenzyme A in the samples were quantified automatically and exported to Excel. Ten compounds, including malonyl coenzyme A (malonyl-CoA), methylmalonyl coenzyme A (methylmalonyl-CoA), succinyl coenzyme A (succinyl-CoA), acetyl coenzyme A (acetyl-CoA), 3-dephospho coenzyme A (3-dephospho-CoA), propionyl coenzyme A (propionyl-CoA), crotonyl coenzyme A (crotonyl-CoA), butyryl coenzyme A (butyryl-CoA), 2-methylcrotonyl coenzyme A (2-methylcrotonyl-CoA), and acetoacetyl coenzyme A (acetoacetyl-CoA) were detected and quantified in the heart tissue samples of rats.

### Molecular dynamic simulation

Molecular dynamic simulations were performed using Gromacs software (version 2019.03). The initial conformation of the simulation involved docking mal-NAC with VDAC2. The Amber-99SB force field was selected for the simulation, and VDAC2 was placed in a water box with TIP3P model. The system was made electrically neutral by randomly adding Na^+^ or Cl^-^ ions, with the shortest distance of VDAC2 protein from the box boundary set to 1 nm. The energy of the system was minimized using the steepest descent method. Balanced NVT and NPT ensembles were set for 500 ps each to reach the preset temperature (300K) and pressure (1 MPa). A 100-ns full atom dynamic simulation was then performed, with all bonds constrained using the UNCS algorithm. The PME method was applied to calculate the electrostatic effect, with a cutoff radius of 1.4 nm for van der Waals force. Isotropic pressure control, Parrinello Rahman pressure bath, and V-rescale temperature control were selected. Data was saved every 10 ps, with simulation steps of 2 fs, and the simulation was run three times in parallel. After the simulation, gmx was used to extract the root mean square deviation (RMSD) of mal-NAC and VDAC2, while root mean square fluctuation (RMSF) was analyzed for VDAC2 protein residues, changes in secondary structure, and the surface area of protein solvent accessibility, with a data point selected every 100 ps. The results were visualized using Origin 8.0 software.

### Circular dichroism

Samples with different concentrations were prepared and analyzed by a Jasco J-1500 circular dichroic spectrometer. The spectrum resolution was set to 0.1nm, with the scanning range from 180nm to 340nm and a scanning rate of 100nm/min. The mean residue ellipticity represented the circular dichroism. The far ultraviolet range scan (190~260nm) revealed characteristic peaks of the protein secondary structure. The proportions of helix, sheet, turn, and random coil of the secondary structure in different wavelength ranges were calculated using the CD Multivariate SSE mode of the Spectra Manager software.

### Synthesis of nano material TPP-AAV(AAV(K46Q)@TPP-PEG@QDs)

Triphenylphosphine (TPP) (10 mmol) and 6-bromohexanoic acid (10.5 mmol) were dissolved in anhydrous acetonitrile and refluxed for 16 hours under nitrogen protection. The resultant was recrystallized to obtain TPP-COOH. Subsequently, TPP-COOH (1 mmol), N, N-dicyclohexylcarbodiimide (1.2 mmol), and N-hydroxysuccinimide (NHS) (1.2 mmol) were dissolved in 5 ml anhydrous DMSO and reacted at room temperature for 12 hours, after which carbon quantum dots (QDs) (4.8 mmol) were introduced to continue the reaction. After 12 hours, the reaction mixture was transferred into a dialysis bag (with a cut-off molecular weight of 1000) and dialyzed with DMSO for 24 hours and deionized water for 48 hours. Thereafter, the dialysate was freeze-dried to obtain TPP-PEG@QDs. TPP-PEG@QDs solution was gently mixed with AAV(K46Q) in a 2:1 ratio, followed by vortexing for 60 seconds, and reaction under nitrogen protection for 60 minutes to form a TPP-PEG@QDs/AAV(K46Q) complex (AAV(K46Q)@TPP-PEG@QDs, TPP-AAV). The particle size analysis was conducted using a laser particle size analyzer (Zetasizer Nano ZS90), while NMR detection was performed through a nuclear magnetic resonance spectrometer (Bruker Avance III 600M).

### Statistical analysis

Statistical analysis was performed using SPSS version 20.0 and GraphPad Prism version 8.0. Data were presented as means ± standard deviation (SD). For animal data, experiments were repeated independently at least six times, while cell data were repeated independently at least three times. Differences between two groups were analyzed using independent sample t-test. Experiments with more than two groups were analyzed using one-way analysis of variance (ANOVA), followed by Tukey's post hoc analysis and (SNK/LSD) comparison. Statistical significance was considered at P < 0.05.

## Results

### VDAC2 malonylation occurred in sepsis-induced myocardial dysfunction

**Figure 1:**
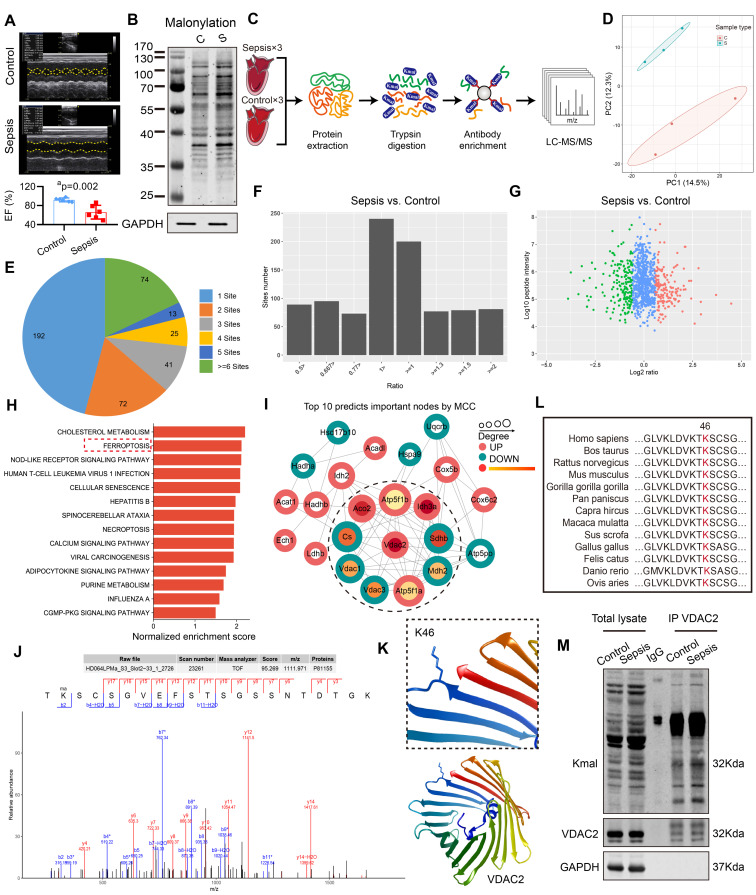
** Enrichment and identification of heart lysine malonylome by Label-Free quantitation. (A)** Cardiac EF of sepsis rats measured by echocardiography (n=6 per group). **(B)** Representative immunoblot of lysine malonylated (Kmal) proteins in control and sepsis groups. GAPDH was used as loading control (n=3 independent experiments). **(C)** Workflow of the strategy for the malonylome analysis. **(D)** Principal components analysis (PCA) score plot of control and sepsis groups. **(E)** Pie chart showing the distribution of the number of identified Kmal sites per protein. **(F)** Histogram showing the ratio distribution of quantifiable Kmal sites between control and sepsis groups. **(G)** Scatterplot showing the quantification of Kmal sites in relation to peptide intensities. **(H)** Pathways enriched by Kyoto Encyclopedia of Genes and Genomes (KEGG). **(I)** MCC analysis of malonylated proteins. The malonylated protein was represented by a concentric circle, with the outer ring color indicating the protein undergoes malonylation up or down, red indicating upregulation, and green indicating downregulation. The redder the inner ring color, the higher the importance of the protein, and the larger the shape, the more interacting proteins there are. **(J)** MS spectrum of the malonylated site Lys46.** (K)** Crystal structure of VDAC2. **(L)** VDAC2 K46 is evolutionarily conserved. The sequences of VDAC2 in thirteen species were aligned. Lysine 46 of VDAC2 was highlighted in red. **(M)** Representative western blots of immunoprecipitated (IP) endogenous malonylated proteins or VDAC2 and immunoblotted for the reciprocal proteins in control and sepsis groups. GAPDH was used as loading control (n=3 independent experiments). The results were analyzed by independent sample t-test. a: p<0.05 as compared with the control group.

The rats in the control group underwent laparotomy without ligation and puncture, and the sepsis group underwent CLP without any intervention. The results of the echocardiography indicated a significant reduction in the left ventricular ejection fraction (LVEF) of sepsis rats (**Figure 1A**), and at the same time the lysine malonylation (Kmal) level was elevated in rat heart tissue after sepsis (**Figure 1B**). Recent studies reported that malonylation is a novel non-enzymatic post-translational modification (PTM) capable of modulating protein structure and function^26^. To identify malonylated proteins, a proteomics screening using the anti-Kmal antibody was performed (**Figure 1C-D**). A total of 1469 Kmal sites across 417 proteins were identified, among them, 1004 Kmal sites from 295 proteins were quantified. Among these Kmal proteins, 192 (46.0%) had a single Kmal site and 74 (17.7%) had more than six Kmal sites (**Figure 1E**). The cut-off ratio for significant Kmal changes between sepsis and control group was set to above 1.5 or below 0.67, and 51 Kmal sites in 28 proteins were up-regulated in sepsis group (**Figure 1F-G**). Enrichment analysis showed that the differentially-expressed (DE) Kmal proteins are mainly associated with ferroptosis and cholesterol metabolism pathways (**Figure 1H**). Using the MCC algorithm, the voltage-dependent anion channel 2 (VDAC2) was predicted as the hub protein among the identified Kmal proteins. It is a membrane-spanning channel that facilitates the transmembrane transport of ions and metabolites (**Figure 1I**). Moreover, the malonylated lysine residue (lys46) of VDAC2 was also identified, and the characteristic tandem mass spectrometry (MS/MS) spectrum including C-terminal y-ions and amino-terminal b-ions was displayed (**Figure 1J-K**). Lys46 (K46) of VDAC2 was found to be highly conserved in different species, ranging from Human to Sheep (**Figure 1L**), and the immunoblotting results (**Figure 1M**) confirmed that malonylated VDAC2 levels were increased in sepsis as compared with control. These results showed that VDAC2 malonylation occurred in SIMD.

### VDAC2 malonylation inducing myocardial injury is through mitochondrial-related ferroptosis after sepsis

**Figure 2:**
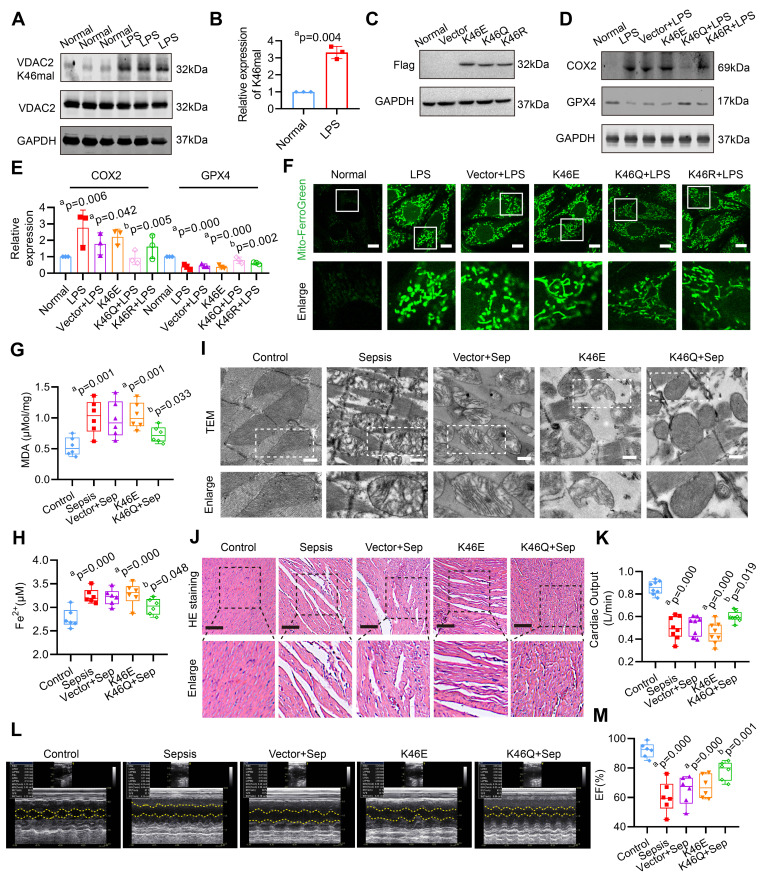
** Role of VDAC2 lysine 46 in the regulation of ferroptosis and myocardial injury. (A)** Western blot analysis and **(B)** relative expression of VDAC2 K46 mal in the H9C2 cells after treatment with LPS (n=3 independent experiments). **(C)** Western blot analysis of Flag (K46E, K46Q and K46R) expression in the H9C2 cells (n=3 independent experiments).** (D-E)** Western blot analysis of GPX4 and COX2 expression in the H9C2 cells after mutating K46 of VDAC2 (n=3 independent experiments). **(F)** Representative images of Mito-FerroGreen (Bar=10μm) (n=3 independent experiments).** (G)** The levels of MDA in rat heart tissue treated with AAV(K46E) and AAV(K46Q) (n=6 each group). **(H)** The levels of Fe^2+^ in rat heart tissue treated with AAV(K46E) and AAV(K46Q) (n=6 each group).** (I)** Representative TEM images of mitochondria in cardiomyocytes of rat heart tissue (Bar=0.5μm) (n=6 each group). **(J)** HE staining of heart tissues of rats (Bar=20μm) (n=6 each group). **(K)** Cardiac output (CO) of rats (n=8 each group). (**L**) Representative echocardiograms images and (**M**) quantitative results of cardiac EF of rats (n=6 per group). The results were analyzed by independent sample t-test and one-way ANOVA. a: p<0.05 as compared with the normal or control group, b: p<0.05 as compared with the LPS or sepsis group.

The site-specific modified antibody was utilized to confirm the malonylation modification of VDAC2 K46. **Figure 2A-B** confirms a significant increase in the malonylation of VDAC2 K46 in H9C2 cells after LPS stimulation. To determine whether regulation of VDAC2 K46 malonylation contributed to the ferroptosis after sepsis, VDAC2 mutants were generated, including K46E (lysine to glutamic acid) that mimic constitutive malonylation, K46Q (lysine to glutamine) and K46R (lysine to arginine) (the 2 mutants were unable to undergo malonylation) and adeno-associated virus (AAV) (K46E, K46Q, and K46R) with the heart specific promoter cTNT were generated. The rats were injected with the corresponding AAVs through the tail vein (1.5×10^12^ vg per rat) 28 days before CLP modeling (**Supplementary Figure 1A-C**). As shown in the **Figure 2C**, the 3 types of VDAC2 mutants were overexpressed in H9C2 cells. LPS stimulation and overexpression of VDAC2 K46E significantly reduced the levels of glutathione peroxidase 4 (GPX4), mito-Fe^2+^ (Mito-Ferro Green), and glutathione/oxidized glutathione (GSH/GSSG), while increasing the levels of cyclooxygenase 2 (COX2) and malondialdehyde (MDA) in H9C2 cells (**Figure 2D-F**, **Supplementary Figure 2**). K46Q and K46R alleviated the mitochondrial-related ferroptosis of H9C2 cells, and the effect of K46Q was stronger than that of K46R (**Figure 2D-F, Supplementary Figure 2**). After injection of AAV (K46E) or in the sepsis group, the levels of MDA and Fe^2+^ were significantly increased as compared with the control group, and down-regulation of VDAC2 malonylation by AAV (K46Q) decreased the levels of MDA and Fe^2+^ of heart tissues in sepsis rats (**Figure 2G-H**). As observed with a TEM, the mitochondrial of heart tissues in sepsis rats and K46E groups presented fragmentation with loss of cristae, which was the hallmark of ferroptosis, while K46Q significantly improved the mitochondrial morphology (**Figure 2I**). The myocardial fibers in sepsis and K46E groups showed obvious disorder with severe swollen myocardial cells and widened muscle gap, and the cardiac output (CO) and LVEF was significantly decreased, while K46Q significantly restored the disorder of myocardial fiber arrangement and increased the cardiac output and LVEF after sepsis (**Figure 2J-M**).

In order to further testify the effects of VDAC2 malonylation on cardiomyocytes ferroptosis, Sirtuin 5 (SIRT5), which was the most important demalonylation enzyme, was overexpressed in H9C2 cells by transfected with Ad-Sirt5 overexpression (OE) (**Supplementary Figure 3A**). As shown in **Supplementary Figure 3B-D**, SIRT5 overexpression significantly decreased the Kmal level and inhibited the ferroptosis of cardiomyocytes. The results indicate that VDAC2 malonylation plays an important role in the occurrence of mitochondrial-related ferroptosis in SIMD.

### The structure change of VDAC2 mediated by VDAC2 lys46 malonylation is the important mechanism of cardiomyocyte ferroptosis

**Figure 3:**
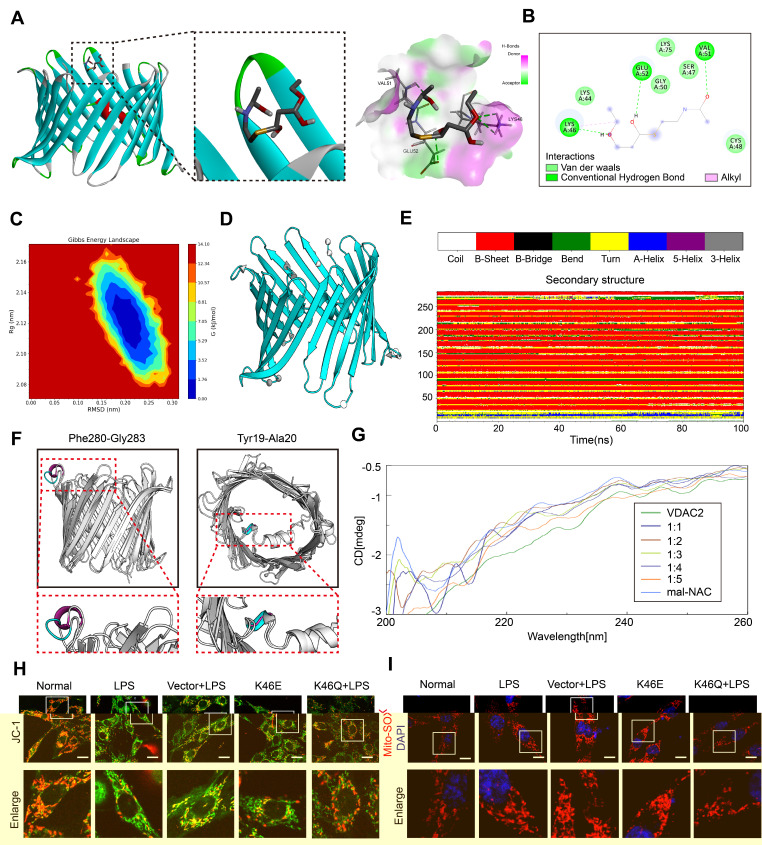
** Molecular dynamics simulation and circular dichroism spectrum of VDAC2. (A)** Molecular docking of VDAC2 and malonyl-CoA.** (B)** Hydrogen bond forming between ligand with lys46 site.** (C)** Gibbs energy landscape.** (D)** Conformational change of VDAC2. **(E-F)** Changes of VDAC2 protein secondary structure over time, with blue representing the initial structure (0ns) and red representing the final structure (100ns). **(G)** Overlay graph of far ultraviolet. **(H)** Mitochondrial membrane potential detected by JC-1(Bar=20μm) (n=3 independent experiments) **(I)** Representative images of Mito-SOX (Bar=10μm) (n=3 independent experiments).

In order to investigate the mechanism of ferroptosis induced by VDAC2 malonylation, we observed the structure changes of VDAC2 after malonylation and its relationship to mitochondrial function. The agonist of malonylation (mal-NAC)^27^ (**Supplementary Figure 4A-B**) was used for molecular dynamics simulation and circular dichroism spectrum analysis. VDAC2 was molecular docked with malonyl group. The results showed that lys46 of VDAC2 could combine with malonyl group to form hydrogen bond and hydrophobic force (π - Alkyl) (**Figure 3A-B**). According to the free energy landscape, the optimal conformation for lys46 site and malonyl group binding was selected (**Figure 3C**). Further 100 ns molecular dynamics simulation was carried out through Gromacs software. By analyzing the three-dimensional structure (**Figure 3D**) at the beginning (0 ns) and end (100 ns) of the simulation, the changes during the binding process of VDAC2 and malonyl group were characterized. The structural changes were presented in the arrow area. The arrow direction pointed to the direction of motion, and the more intensive the arrows were, the more significant the movement was, indicating that the regional structure had obvious volatility. Further analysis according to the DSSP algorithm found that the VDAC2 contained nineteen β-sheets, one β-bend and one α-helix structure. Among them, the Pro265-Thr270 section showed obvious structural transformation during the simulation process, and the adjacently arranged β-turn and β-bend were transformed into partial 5-helix and α-helix at about 30-60 ns, and then tended to form a continuous β-turn, and after 85 ns, it gradually changes into a state of alternating β-turn and β-bend. The Glu212-Val218 section exhibited frequent inter-transitions of β-turn and β-bend during simulation (**Figure 3E**). The comparison of the secondary structure at the initial (0 ns) and end (100 ns) revealed 2 structural changes, Phe280-Gly283 (random coil changed to α-helix) and Tyr19-Ala20 (α-helix changed to random coil) (**Figure 3F**).

Further, VDAC2 (1mM) were treated with malonyl-NAC at different proportions. Through analyzing by circular dichroism, the secondary structure of VDAC2 changed significantly after treatment with malonyl-NAC, mainly manifested as helix decreased and random coil increased (**Figure 3G, Supplementary Table 1**). As a mitochondrial outer membrane channel protein, VDAC2 structural change was closely related to mitochondrial function. Thus, the mitochondrial membrane potential (MMP) and mitochondrial ROS were observed through JC-1 and Mito-Sox *in vitro*. The results showed that the MMP of H9C2 cells after LPS stimulation was significantly reduced, and mitochondrial ROS was significantly increased. Overexpressed K46E had a similar result to the LPS group. By contrast, overexpressed K46Q significantly improved the mitochondrial function, increased the MMP, and reduced the level of mitochondrial ROS (**Figure 3H-I, Supplementary Figure 4C-D**). These results suggested that VDAC2 malonylation could result in the structure change of VDAC2, and affect the MMP and ROS production of mitochondria, and finally induced the ferroptosis of cardiomyocytes.

### The increase of malonyl-CoA is the reason of VDAC2 malonylation after sepsis

**Figure 4:**
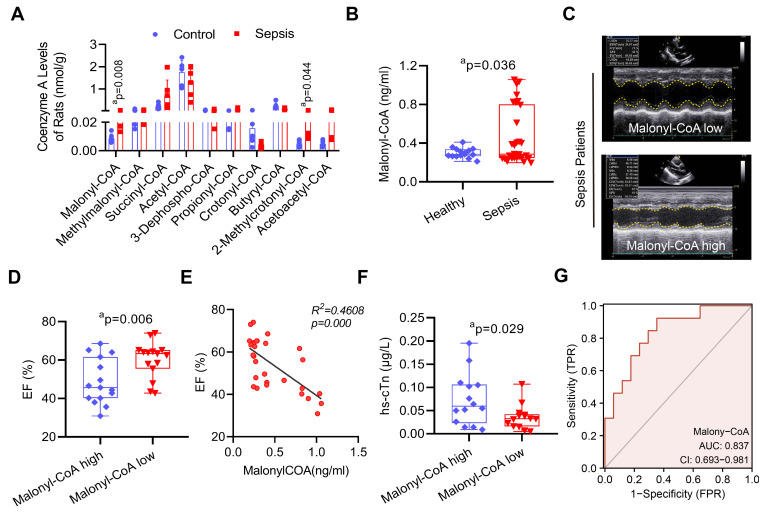
**The role of malonyl-CoA in myocardial injury after sepsis. (A)** Detection of myocardial acyl-coenzyme A level in rats using targeted metabolomics. Acyl-coenzyme A levels were compared between control and sepsis groups (n=6 each group). **(B)** Comparison of serum malonyl-CoA levels in healthy control and sepsis patients. **(C)** Representative echocardiogram images of sepsis patients.** (D)** LVEF of sepsis patients in the serum malonyl-CoA high and low groups.** (E)** Person's correlation analysis between LVEF and serum malonyl-CoA of sepsis patients. **(F)** hs-cTn levels in sepsis patients.** (G)** ROC curve for serum malonyl-CoA levels in sepsis patients. The results were analyzed by independent sample t-test. a: p<0.05 as compared with the control or malonyl-CoA high group.

To investigate the underlying mechanism of sepsis-induced VDAC2 malonylation. LC-MS-based metabolomics was applied to detect the level of myocardial malonyl-CoA (**Supplementary Figure 5**). The result showed that as compared with the control group, the malonyl-CoA and 2-methylrotonyl-CoA of heart tissues in the sepsis rats were significantly increased (**Figure 4A**). To further verify the role of malonyl-CoA in myocardial injury in sepsis patients, the serum malonyl-CoA levels were detected in 30 sepsis patients and 15 healthy controls (basic information of them were shown in **Supplementary Table 2**). The serum level of malonyl-CoA in sepsis patients was significantly higher than that in the healthy controls (**Figure 4B**). Then the sepsis patients were divided into the high-malonyl-CoA and low-malonyl-CoA subgroups according to the median value (0.287ng/ml) of malonyl-CoA (clinical information of the two groups was shown in **Supplementary Table 3**). The results of echocardiography showed that the LVEF of patients was significantly lower in high-malonyl-CoA group (**Figure 4C-D**), and correlation analysis showed that LVEF was negatively correlated with malonyl-CoA, R^2^ = 0.4608 (**Figure 4E**). Besides, the levels of hs-cTn between the two groups was also significantly different (**Figure 4F**). ROC analysis showed that the cut-off value of malonyl-CoA was 0.272ng/ml and the AUC was 0.837 (**Figure 4G**). The above results indicated that malonyl-CoA played an important role in the occurrence of SIMD.

The myocardial proteomic analysis was also performed to explore the mechanism of the up-regulation of malonyl-CoA in sepsis rats (**Supplementary Figure 6A**). As shown in the **Supplementary Figure 6B-C**, there were 339 differentially expressed (DE) proteins upregulated and 2108 DE proteins downregulated in sepsis rats (a cutoff of unique peptide≥1; fold change (FC) > 1.5 or ≤ 0.67; and P-value < 0.05). The DE proteins were remarkably concentrated in metabolic pathways such as Glycolysis/Gluconeogenesis, Glutathione metabolism, and Fatty acid metabolism (**Supplementary Figure 6D**). The heatmap and western blot showed that the expressions of carnitine palmitoyl transterase-1 (CPT1), and phosphorylation of acetyl-CoA carboxylase (p-ACC2), and malonyl-CoA decarboxylase (Mlycd/MCD) decreased significantly after sepsis (**Supplementary Figure 6E-F**). These results indicated that malonyl-CoA was increased in heart tissues after sepsis.

### Inhibition of Malonyl-CoA had a protective effect against mitochondrial-related ferroptosis in SIMD

**Figure 5:**
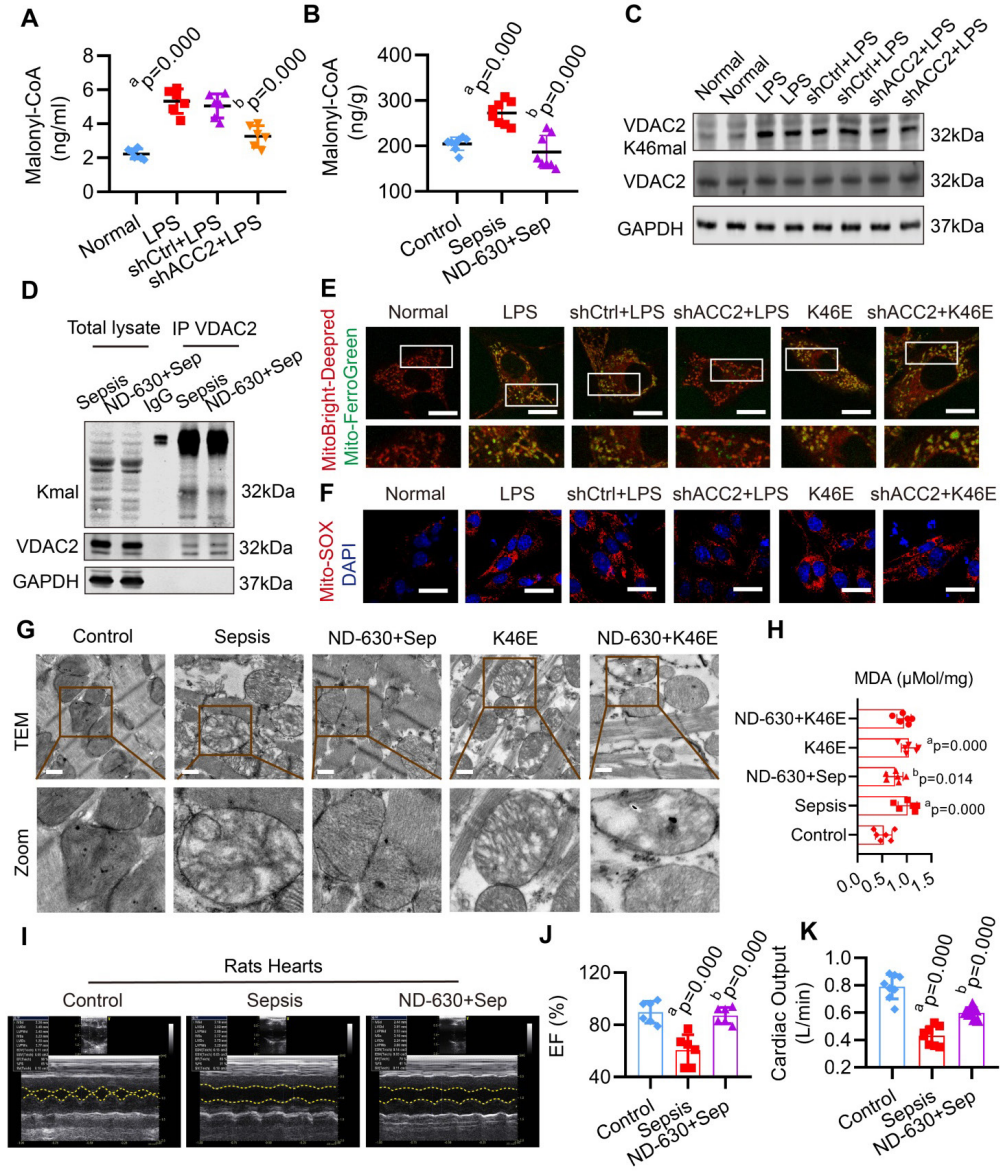
** Effect of malonyl-CoA on VDAC2 malonylation and ferroptosis in sepsis myocardial injury.** The malonyl-CoA levels were measured in** (A)** H9C2 cells (n=3 independent experiments) and **(B)** heart tissues (n=8 each group) detected using ELISA. **(C)** Western blot analysis was performed to determine the expression of VDAC2 K46mal in H9C2 cells after transfection with Ad-Vector and Ad-shACC2 (n=3 independent experiments). **(D)** Representative western blots of immunoprecipitated (IP) endogenous malonylated proteins or VDAC2 and immunoblotted for the reciprocal proteins in sepsis and ND-630 groups (n=3 independent experiments).** (E)** Representative images of H9C2 cells after treatment with MitoBright-Deep Red and Mito-FerroGreen (Bar=5μm) (n=3 independent experiments).** (F)** Representative images of Mito-SOX in H9C2 cells (Bar=10μm) (n=3 independent experiments). **(G)** Representative TEM images of mitochondria in cardiomyocytes of rat heart tissue (Bar=0.5μm) (n=6 each group).** (H)** The level of MDA (n=6 independent experiments).** (I)** Echocardiograms images of rats (n=6 each group).** (J)** Summary of cardiac EF of rats measured by echocardiography.** (K)** Cardiac output (CO) of rats (n=8 each group). The results were analyzed by one-way ANOVA. a: p<0.05 as compared with the normal or control group, b: p<0.05 compared with sepsis or LPS group.

In order to explore whether inhibiting malonyl-CoA could down-regulate the malonylation of VDAC2 and decrease the mitochondrial-related ferroptosis in SIMD, acetyl-CoA carboxylase 2 (ACC2) interfering adenovirus (Ad-shACC2) (**Supplementary Figure 7A-B**) and ACC2 inhibitor (ND-630) were used to cut down the level of malonyl-CoA and VDAC2 malonylation *in vivo* and vitro (**Figure 5A-D**, **Supplementary Figure 7C**). Compared with the LPS group, Ad-shACC2 could decrease the level of mito-Fe^2+^, mito-ROS, and MDA, and increase the level of GSH/GSSG (**Figure 5E-F, Supplementary Figure 8**). However, Ad-shACC2 transfection could not abolish K46E induced ferroptosis of H9C2 cells (**Figure 5E-F, Supplementary Figure 8**). The rats in the ND-630 group were treated with ND-630 (3 mg/kg, i. v.) 30 min before the CLP. ND-630 could improve the mitochondrial morphology and decrease the level of MDA of cardiomyocytes in heart tissue of sepsis rats (**Figure 5G-H**). The results showed that the mitochondrial morphology and MDA level of K46E treated rats could not been improved by ND-630 (**Figure 5G-H**). Moreover, ND-630 could also effectively ameliorate SIMD by increasing LVEF, CO, and survival rate of sepsis rats (**Figure 5I-K**, **Supplementary Figure 9**). These results indicated that the inhibition of malonyl-CoA could down-regulate VDAC2 malonylation and protect against mitochondrial-related ferroptosis in SIMD.

### Synthesis of VDAC2 malonylation targeting treatment nano material

**Figure 6:**
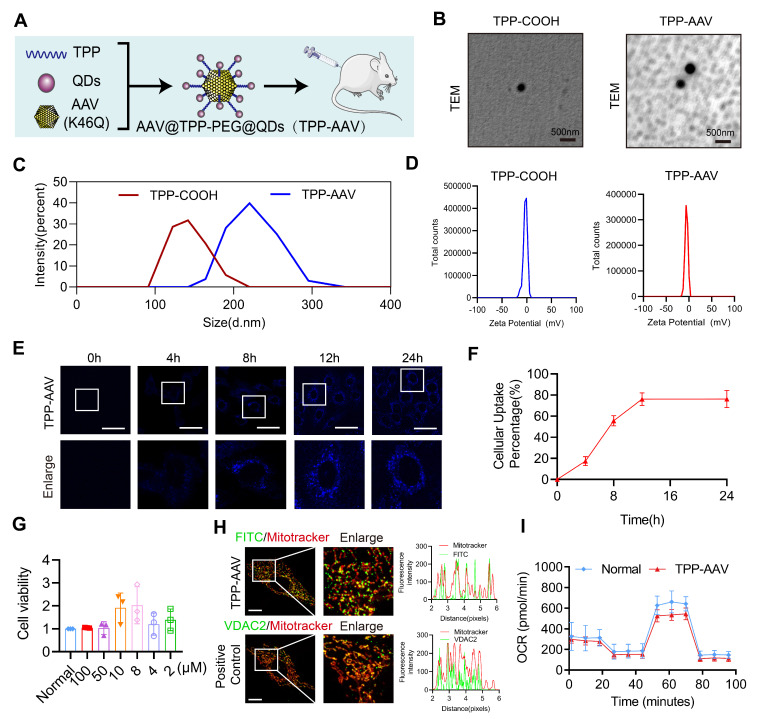
** Characterization of nano material TPP-AAV. (A)** Synthesis flow chat of TPP-AAV. **(B)** Representative TEM images of TPP-COOH and TPP-AAV (Bar=500μm). The **(C)** size and **(D)** zeta potential of TPP-COOH and TPP-AAV. **(E)** Representative confocal images of TPP-AAV at different times (Bar=50μm).** (F)** Relative cellular uptake percentage of TPP-AAV (n=3 independent experiments).** (G)** Effect of TPP-AAV on cell viability (n=3 independent experiments).** (H)** Representative image of TPP-AAV and positive control (VDAC2) mitochondrial colocalization (Bar=10μm) (n=3 independent experiments).** (I)** Mitochondrial respiration in H9C2 cells (n=3 independent experiments). The results were analyzed by independent sample t-test and one-way ANOVA.

VDAC2 as a channel protein at the outer mitochondrial membrane^28^, in order to enhance the therapeutical effect of inhibition of VDAC2 malonylation, a new nano material, TPP-AAV(AAV(K46Q)@TPP-PEG@ODs), which could target mitochondria to inhibit VDAC2 malonylation was synthetized (**Figure 6A**). TPP-COOH 1H NMR features are as δ 12.01 (s, 1H-COOH), 7.91-7.75 (m, 15H, Ar-H), 3.62-3.53 (m, 2H, -CH2-), 2.17-2.14 (m, 2H, -CH2-), 1.5 (s, 6H, -CH2-). PEG 1H NMR features are as δ 2.27-2.25 (m, -NHCH2CH2-). **Supplementary Figure 10A** showed a characteristic chemical shift of TPP- COOH from 7.75 to 7.91, and the characteristic chemical shift of PEG from 2.27 to 2.55. The electronic microscope observation showed that TPP-COOH and TPP-AAV were spherical with regular particles (**Figure 6B**). The average particle size of TPP-COOH and TPP-AAV were (139.0 ± 22.6) and (220.6 ± 29.1) nm (**Figure 6C**), and the zeta potential were (-2.8 ± 4.2) and (-5.5 ± 3.2) MV (**Figure 6D**), respectively. These results indicated that the target material TPP-AAV has been successfully synthesized. Immunofluorescence result showed that the material TPP-AAV was successfully up-taken by H9C2 cells (**Figure 6E-F**).

### The toxicity and mitochondrial targeting of nano material TPP-AAV

In order to evaluate the toxicity of the nano material, we observed the effects of TPP-AAV(AAV(K46Q)@TPP-PEG@ODs) nano material on histology of vital organs of rats and cell viability of H9C2 cells. The results showed that TPP-AAV had no damage effect on the vital organs of rats in histology by HE staining (**Supplementary Figure 10B**). Also, TPP-AAV had no toxicity on H9C2 cells evaluated by CCK-8 assay (**Figure 6G**). Besides, TPP-AAV could effectively transfect H9C2 cells without affecting the normal morphology of cells (**Supplementary Figure 10C**). Mitochondrial localization was an important index to evaluate the targeting of TPP-AAV. The results of immunofluorescence showed that TPP-AAV could co-localize with the mitochondria of cardiomyocytes, indicating that the nano material could target mitochondria (**Figure 6H**), and did not change the oxygen consumption rate (**Figure 6I**).

### The targeting treatment effect on ferroptosis and SIMD of TPP-AAV

**Figure 7:**
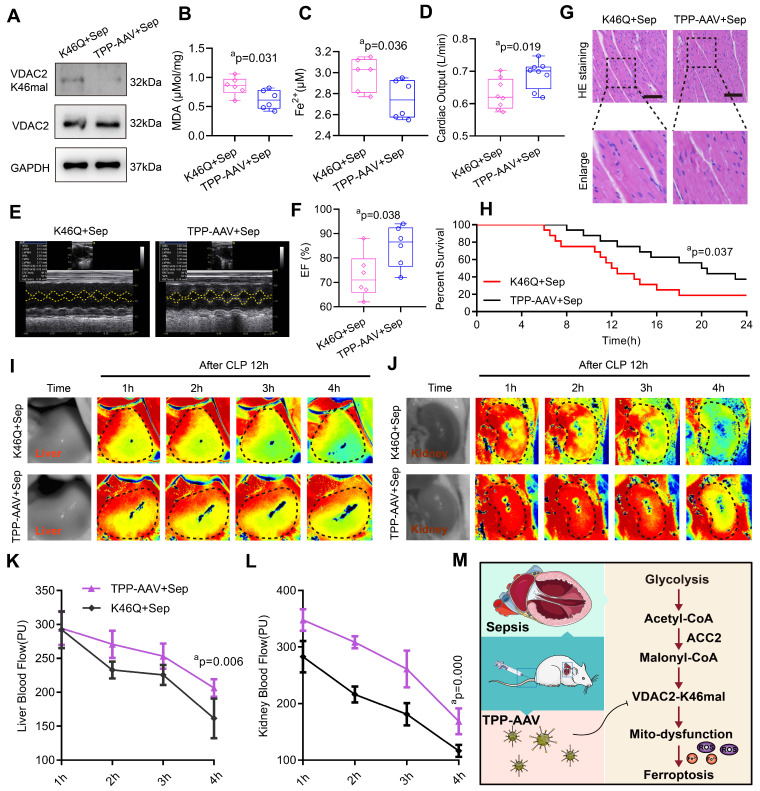
** Protective effects of nano material TPP-AAV on SIMD. (A)** The expression of VDAC2 (K46) malonylation in cardiac tissues of sepsis rats after treatment with AAV(K46) or TPP-AAV (n=3 independent experiments). The level of cardiac **(B)** MDA and **(C)** Fe^2+^ after injection of TPP-AAV in sepsis rats (n=6 each group). **(D)** Cardiac output (CO) (n=8 each group). (**E**) Representative echocardiograms images and (**F**) quantitative results of cardiac EF of sepsis rats after being treated with AAV (K46Q) and TPP-AAV (n=6 each group).** (G)** HE staining of heart tissues after injection of TPP-AAV (Bar=25μm) (n=6 each group).** (H)** Survival rate of sepsis rats after treatment with TPP-AAV (n=16 each group).** (I-L)** Laser speckle technique was used to monitor time-lapse liver and kidney blood flow of rats (n=6 each group).** (M)** Schematic diagram of ferroptosis of cardiomyocytes in sepsis caused by VDAC2 malonylation and targeted treatment by TPP-AAV. The results were analyzed by independent sample t-test. Rat survival was assessed by Kaplan-Meier analysis. a: p<0.05 as compared with the K46Q group.

We observed the treatment effect of TPP-AAV. As compared with AAV (K46Q), TPP-AAV(AAV(K46Q)@TPP-PEG@ODs) significantly decreased the levels of VADC2 (K46) malonylation, cardiac MDA and Fe^2+^ and increased cardiac output (CO) and LVEF of sepsis rats (**Figure 7A-F**). In addition, TPP-AAV restored myocardial fiber arrangement more effectively than AAV (K46Q), as evidenced by HE staining of cardiac pathology (**Figure 7G**). Besides, the survival rate of the K46Q group were 18.75%, and TPP-AAV extended the survival rate to 37.5% (**Figure 7H**). Additionally, liver and kidney blood flow were measured at 1h, 2h, 3h, and 4h after CLP 12h using a laser speckle blood flow imaging instrument. TPP-AAV significantly improved liver and kidney blood perfusion of sepsis rats as compared with AAV (K46Q) (**Figure 7I-L**). The results indicated that TPP-AAV was superior to AAV (K46Q) in reducing ferroptosis and myocardial injury after sepsis by inhibiting VDAC2 malonylation.

## Discussion

Our study revealed that cardiomyocytes display increased VDAC2 malonylation after sepsis. The regulation of VDAC2 malonylation can significantly impact mitochondrial-related ferroptosis and myocardial injury after sepsis. Malonylation of VDAC2 alters its structure, ultimately resulting in mitochondrial dysfunction. The malonyl-CoA was increased in sepsis rats and patients, which can induce VDAC2 malonylation. VDAC2 malonylation targeting nano material TPP-AAV effectively relieved the myocardial injury via inhibiting the ferroptosis of cardiomyocytes (**Figure 7M**).

Ferroptosis is a recently discovered form of programmed cell death that differs from apoptosis, necroptosis, and pyroptosis. Targeting ferroptosis is crucial in treating SIMD. Wang et al. observed that dexmedetomidine could inhibit ferroptosis by regulating HO-1, thus reducing sepsis-induced myocardial injury^23^. Similarly, Zeng et al. demonstrated that resveratrol attenuated SIMD in rats by suppressing ferroptosis via the Sirt1/Nrf2 pathway^29^. In this study, we established, for the first time, a connection between VDAC2 malonylation and ferroptosis, presenting a novel potential mechanism for SIMD.

VDAC2 is a channel protein located at the outer mitochondrial membrane^30^. The dynamic "open-close" of VDAC2 affects the mitochondrial metabolism and cellular bioenergy. When VDAC2 is open, it permits ADP and phosphoric acid substrates to enter mitochondria. However, when VDAC2 is closed, mitochondrial transport function is blocked^31^-^32^. Acetaldehyde, a metabolite of ethanol dehydrogenation, induces VDAC closure, resulting in mitochondrial substrate transport inhibition, ATP production suppression, and mitochondrial depolarization^33^. Nevertheless, continuously opening VDAC2 by erastin increases the permeability of the mitochondrial outer membrane, disturbing mitochondrial metabolism and oxidation functions, causing cellular homeostasis imbalance and increasing ferroptosis^34^. In the present study, we found that lys46 site malonylation induced a conformational change of VDAC2. Consequently, this leads to decreased mitochondrial membrane potential, and an increase in mitochondrial Fe^2+^ and ROS levels, ultimately leading to ferroptosis. The VDAC2 protein contains 294 amino acids, including a N-terminus of 36-amino acid and 19 β strands. The N-terminus situated in the lumen of the transmembrane β-barrel, regulates channel opening and closing^35^-^36^. Our molecular dynamics simulation of VDAC2 observed changes in the secondary structure of Tyr19-Ala20 (this region belonged to N-terminal structure), indicating that the N-terminal structure is involved in malonylation-induced conformational changes. Our findings suggest that changes in VDAC2 conformation affect mitochondrial metabolism and mitochondrial-related ferroptosis, potentially aggravating SIMD. VDAC2 may be an important therapeutic target for SIMD, and future studies will explore the underlying mechanisms.

This study aimed to synthesize a targeting treatment nano material called TPP-AAV for VDAC2 malonylation inhibition. Adeno-associated virus (AAV) vectors have been utilized as the most frequent delivery system for *in vivo* gene therapy in at least 136 unique human clinical trials to treat 55 different disease indications^37^. In order to improve the therapeutic effect of AAV(K46Q), the triphenyl-phosphonium bromide (TPP) was used to target mitochondria. TPP is a lipophilic cation which is capable of targeting mitochondria by taking advantage of the negative membrane potential of mitochondria^38^. Modification of carbon quantum dots can reduce the trace toxicities in TPP and enhance its ability to penetrate the membrane^38^. This increases mitochondrial accumulation, prolongs the biological activity of AAV, and results in better efficiency for targeting and improved bioavailability^39^. Our results indicated that TPP-AAV was effective in targeting the mitochondria and achieved reduction in ferroptosis, in addition to improvement in cardiac function, and cause of the survival rate increase in sepsis rats. Targeted therapy on protein post-translational modification exhibited great potential and broad application prospects, which needs further research in the future.

Malonyl-CoA, the precursor of fatty acid synthesis, primarily exists in mitochondria, peroxisomes, and cytoplasm. It serves as a negative mediator of fatty acid oxidation by inhibiting CPT-1 and blocking fatty acids' entry into the mitochondria^40^. Additionally, malonyl-CoA can modulate various protein functions through malonylation - an evolutionarily conserved post-translational modification (PTM) process. Malonylation involves adding a malonyl group to a protein lysine residue, which shifts its charge from +1 to -1 and may disrupt electrostatic interactions with other amino acids. This process can alter protein conformation, affect protein activity and functions^41^-^42^. We discovered for the first time that malonyl-CoA upregulation after sepsis can increase VDAC2 malonylation. Aside from VDAC2, we also observed malonylation of cytoskeleton-related proteins, troponin, and tricarboxylic acid cycle enzymes. However, their specific roles require further investigation.

The malonyl-CoA level in cells is mainly regulated by acetyl-CoA carboxylase (ACC) and malonyl-CoA decarboxylase (MCD)^43^. In mammals, there are two tissue-specific isoenzymes of ACC: ACC1 found in adipose tissue and liver, and ACC2 found in liver, heart, and skeletal muscles^44^-^45^. The main function of ACC2 is to convert acetyl-CoA to malonyl-CoA, and the activity of ACC2 is affected by many factors, such as the allosteric effect of citrate, the present of long-chain acyl-CoA, and the phosphorylation of PKA or AMPK^46^. LPS-stimulated macrophages can enhance glycolysis and inhibit the tricarboxylic acid cycle (TCA). As a result, metabolic intermediates such as citrate and succinate accumulate in mitochondria^47^. In sepsis, cardiomyocytes shift from fatty acid oxidation to glycolysis, leading to citrate accumulation, which is an essential allosteric activator of ACC2^48^. Subsequently, the amassed citrate undergoes citrate-pyruvate cycling in the cytoplasm, generating acetyl-CoA that ACC2 activates into malonyl-CoA. Consequently, malonyl-CoA serves as the substrate for malonylation.

In summary, the present study revealed that the malonylation of K46 in VDAC2 is a novel mechanism in sepsis induced myocardial dysfunction. AAV@TPP-PEG@QDs (TPP-AAV) targeted therapy can effectively inhibit the ferroptosis of cardiomyocytes and alleviate myocardial injury after sepsis. These results provide new insights to the mechanism of SIMD and provide a new strategy through targeted technology.

## Supplementary Material

Supplementary figures and tables.

## Abbreviations

AAV: Adeno-associated virus
ACC: acetyl-CoA carboxylase
CLP: cecal ligation and puncture
COX2: cyclooxygenase 2
CPT1: carnitine palmitoyl transterase-1
GPX4: glutathione peroxidase 4
Kmal: lysine malonylation
LPS: lipopolysaccharide
MAP: mean artery pressure
MCD: malonyl-CoA decarboxylase
MDA: malondialdehyde
PTMs: post-translational modifications
QDs: quantum dots
SIMD: sepsis-induced myocardial dysfunction
SIRT5: sirtuin 5
TPP: Triphenyl-phosphonium bromide
VDAC2: voltage-dependent anion channel 2
