# Supplementary material for: VDAC2 malonylation participates in sepsis-induced myocardial dysfunction via mitochondrial-related ferroptosis
Source: Int J Biol Sci. 2023 Jun 14;19(10):3143–58. doi: 10.7150/ijbs.84613 (PMC10321281; doi:10.7150/ijbs.84613)
Supplement: Supplementary file 1 — Supplementary figures and tables. [file ijbsv19p3143s1.pdf]

VDAC2 malonylation participates in sepsis-induced myocardial dysfunction via mitochondrial-related ferroptosis

Han She[1,2†], Lei Tan[1,2†], Yuanlin Du[2†], Yuanqun Zhou[1], Ningke Guo[1], Jun Zhang[2], Yunxia Du[1,2], Yi Wang[1,2], Zhengbin Wu[3], Chunhua Ma[1], Qinghui Li[1], Qingxiang Mao[2], Yi Hu[2*], Liangming Liu[1*] and Tao Li[1*]

[1]State Key Laboratory of Trauma, Burns and Combined Injury, Shock and Transfusion Department, Daping Hospital, Army Medical University, Chongqing400042, China

[2]Department of Anesthesiology, Daping Hospital, Army Medical University, Chongqing400042, China

[3]Department of Intensive care unit, Daping Hospital, Army Medical University, Chongqing400042, China

[*]**Corresponding Authors:**

[*]Tao Li, [*]Liangming Liu and [*]Yi Hu

E-mail: lt200132@163.com, lmliu62@tmmu.edu.cn, huyi921@sina.com

Tel: +86-23-68757525

Fax: +86-23-68757525

[†]These authors contributed equally to this work

Supplementary Tables

Supplementary Table 1. Calculation and analysis results of secondary structural feature of VDAC2.

**Sample**	**Helix**	**Sheet**	**Turn**	**Random coil**
VDAC2	12.4%	30.3%	14.8%	42.4%
VDAC2: mal-NAC (1:1)	10.4%	31.9%	14.2%	43.4%
VDAC2: mal-NAC (1:2)	14.4%	28.0%	14.6%	42.9%
VDAC2: mal-NAC (1:3)	5.8%	34.7%	14.0%	45.4%
VDAC2: mal-NAC (1:4)	10.8%	32.4%	13.6%	43.1%
VDAC2: mal-NAC (1:5)	11.9%	26.4%	14.5%	47.1%

Supplementary Table 2. Baseline characteristics of the sepsis patients and healthy controls in this study.

**Characteristics**		**Sepsis patients (n=30)**	**Healthy controls (n=15)**	**P-Value**
Age, yrs	Mean	60.1	50.4	0.053
	SD	14.5	17.2	
Gender, n (%)	Female	10 (33.3)	6 (40.0)	0.660
	Male	20 (66.7)	9 (60.0)	
Temperature, ℃	Mean	36.9	36.5	0.010
	SD	0.5	0.3	
White blood cell count, $\times 10^9$/L	Mean	16.9	6.6	0.003
	SD	12.2	3.1	
Heart rate/min	Mean	94.9	79.1	0.001
	SD	15.3	8.3	
Severe pneumonia, n (%)	Yes	3 (10.0)	0	0.540
	No	27 (90.0)	15 (100.0)	
Multiple injuries, n (%)	Yes	2(6.7)	0	0.546
	No	28 (93.3)	15 (100.0)	
Pancreatitis, n (%)	Yes	4 (13.3)	0	0.285
	No	26 (86.7)	15 (100.0)	

Supplementary Table 3. Baseline characteristics of the high-malonyl-CoA and low-malonyl-CoA groups in this study.

**Characteristics**		**high-malonyl-CoA (n=15)**	**low-malonyl-CoA (n=15)**	**P-Value**
Age, yrs	Mean	60.2	59.9	0.961
	SD	14.4	15.1	
Gender, n (%)	Female	5 (33.3)	5 (33.3)	> 0.999
	Male	10 (66.7)	10 (66.7)	
Temperature, °C	Mean	36.8	37.0	0.503
	SD	0.5	0.6	
White blood cell count, $\times 10^9$/L	Mean	15.0	18.9	0.387
	SD	8.3	15.3	
Heart rate/min	Mean	95.5	94.3	0.843
	SD	16.2	14.9	

Supplementary Figures and Legends

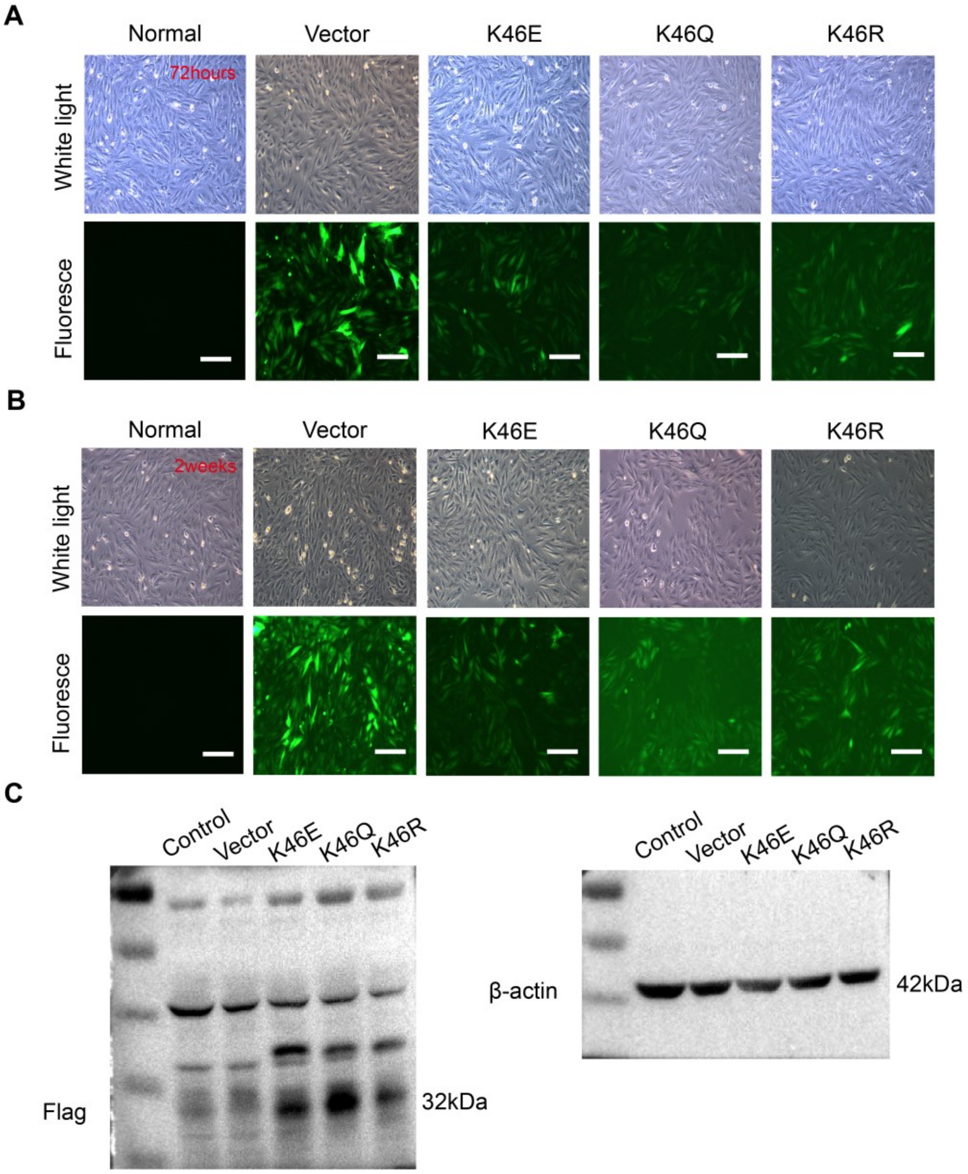

Supplementary Figure 1. Representative fluorescence photos of H9C2 cells after transfection with lentivirus for **(A)** 72hours or **(B)** 2 weeks (Bar=40μm). **(C)** Western blot analysis was performed to determine the expression of Flag (K46E, K46Q and K46R) in the heart tissues of rats after treatment with AAV (K46E, K46Q and K46R) (n=3 independent experiments).

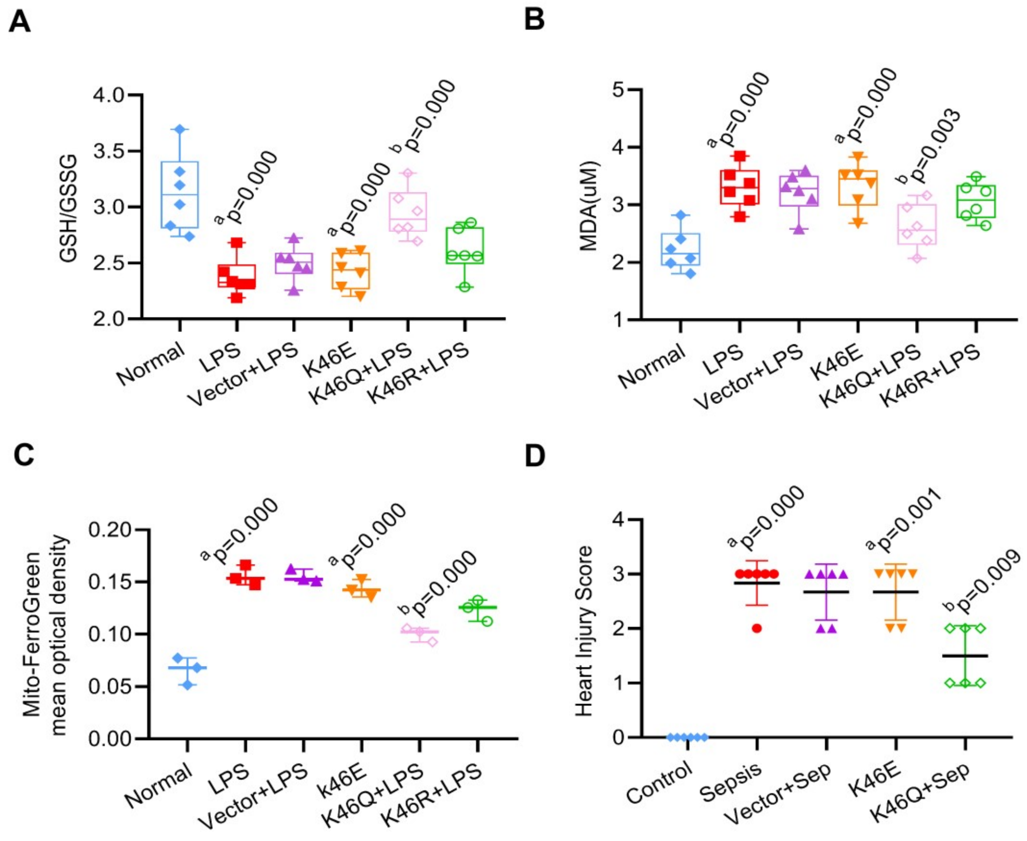

Supplementary Figure 2. The level of **(A)** GSH/GSSG and **(B)** MDA of H9C2 cells after mutating of VDAC2 K46 (n=3 independent experiments). **(C)** The mean optical density of Mito-FerroGreen (n=3 independent experiments). **(D)** The heart injury score of rats (n=6 each group). The results were analyzed by one-way ANOVA. a: $p < 0.05$ as compared with the normal or control group. b: $p < 0.05$ as compared with the LPS or sepsis group.

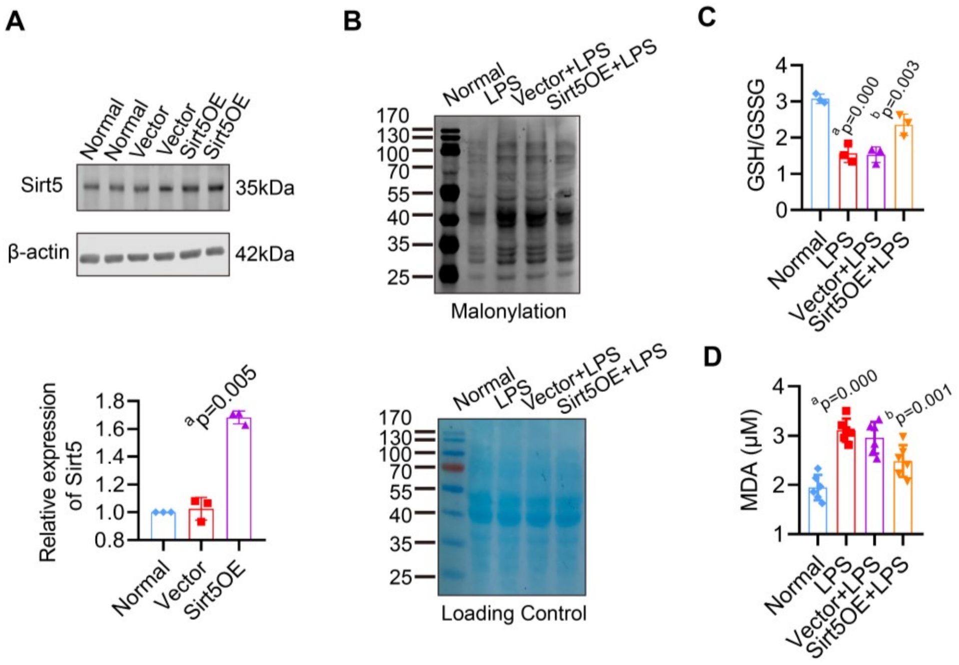

Supplementary Figure 3. The transfection and effects of adenovirus Sirt5 overexpression (Sirt5 OE). (A) Western blot analysis and relative expression of Sirt5 in H9C2 cells after transfected with Ad-Vector and Ad-Sirt5 OE (n=3 independent experiments). **(B)** Western blot analysis of lysates from H9C2 cells by anti-malonyl-lysine antibody. Equal loading was verified using coomassie blue staining (n=3 independent experiments). **(C)** The level of GSH/GSSG in H9C2 cells (n=3 independent experiments). **(D)** The level of MDA in H9C2 cells (n=6 independent experiments). The results were analyzed by one-way ANOVA. a: $p < 0.05$ as compared with the normal group. b: $p < 0.05$ as compared with the LPS group.

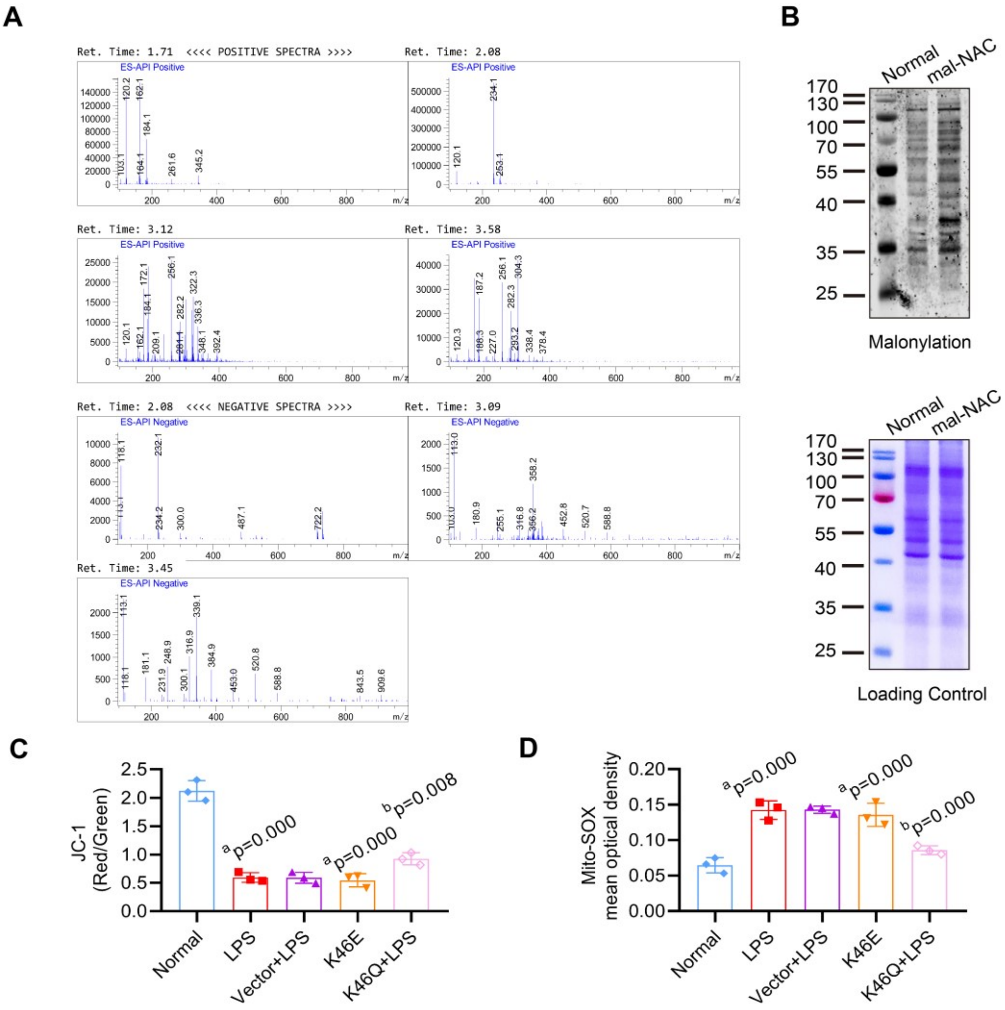

Supplementary Figure 4. (A) Mass spectrometry of mal-NAC. **(B)** The effects of mal-NAC (1mM) on cellular malonylation in H9C2 cells (n=3 independent experiments). (C) Red/green fluorescent quantitative analysis in H9C2 cells (n=3 independent experiments). (D) The mean optical density of Mito-SOX in H9C2 cells (n=3 independent experiments). The results were analyzed by one-way ANOVA. a: $p < 0.05$ as compared with the normal group. b: $p < 0.05$ as compared with the LPS group.

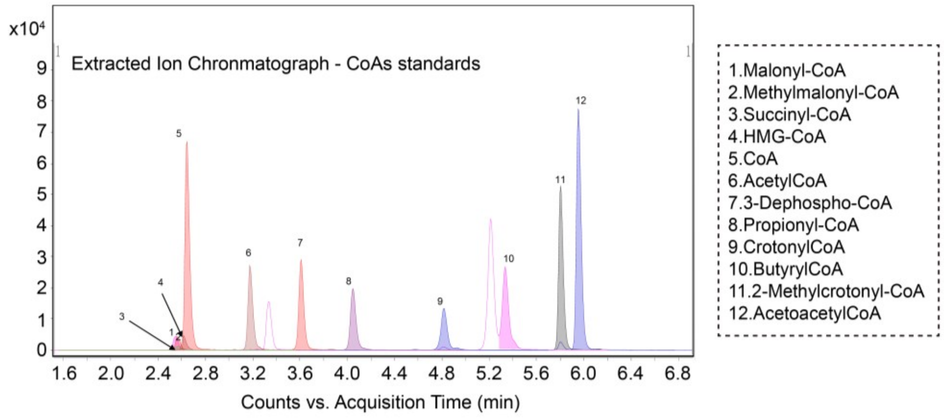

Supplementary Figure 5. Detection of myocardial acyl-coenzyme A level by targeted metabolomics. Extraction of ion chromatograph of coenzyme A standards (n=6 each group).

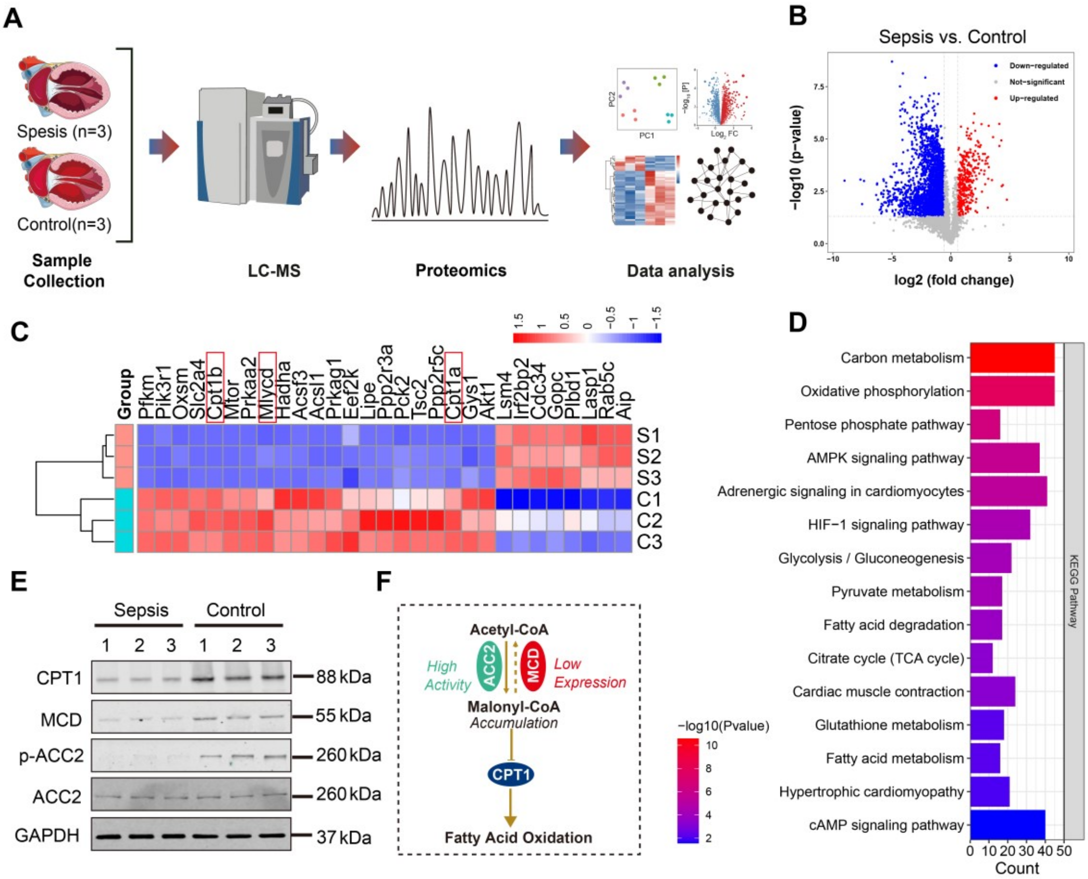

Supplementary Figure 6. Proteomic analysis of myocardial tissues. (A) Schematic diagram of workflow of proteomic analysis. **(B)** Volcano plot analysis of differentially expressed (DE) proteins between control and sepsis group (a cutoff of unique peptide≥1; fold change > 1.5 or ≤ 0.67; and P-value < 0.05). **(C)** Hierarchical clustering heatmap of DE proteins between sepsis and control groups of rats. **(D)** Kyoto Encyclopedia of Genes and Genomes (KEGG). **(E)** The expression of CPT1, MCD, p-ACC2 and ACC2 in myocardial tissues of rats shown by western blot (n=3 independent experiments). **(F)** Schematic diagram showing the changes of malonyl-CoA after sepsis.

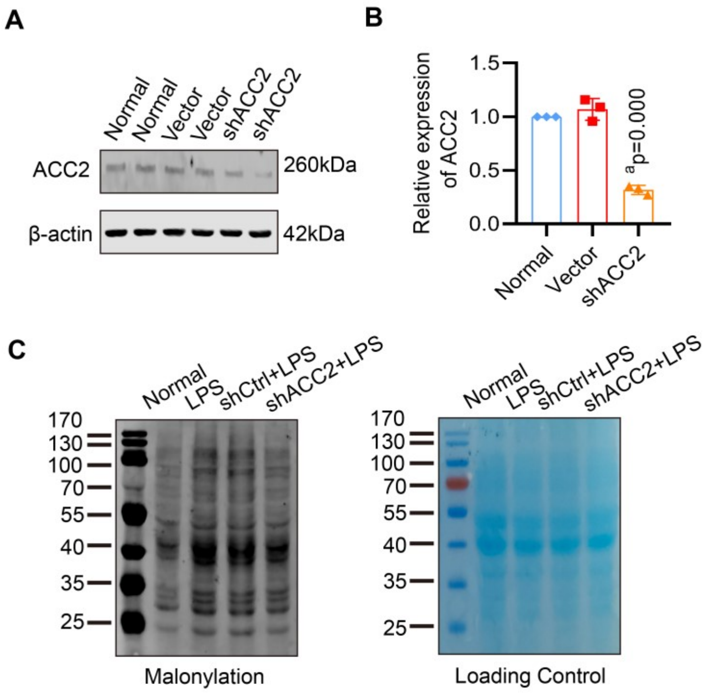

Supplementary Figure 7. The transfection and interference effects of adenovirus scramble ACC2 (shACC2). (A) Western blot analysis and **(B)** relative expression of ACC2 in H9C2 cells after transfected with Ad-Vector and Ad-shACC2 (n=3 independent experiments). **(C)** Western blot analysis of lysates from H9C2 cells by anti-malonyl-lysine antibody. Equal loading was verified using Coomassie blue staining. The results were analyzed by one-way ANOVA. a: $p < 0.05$ as compared with the normal group.

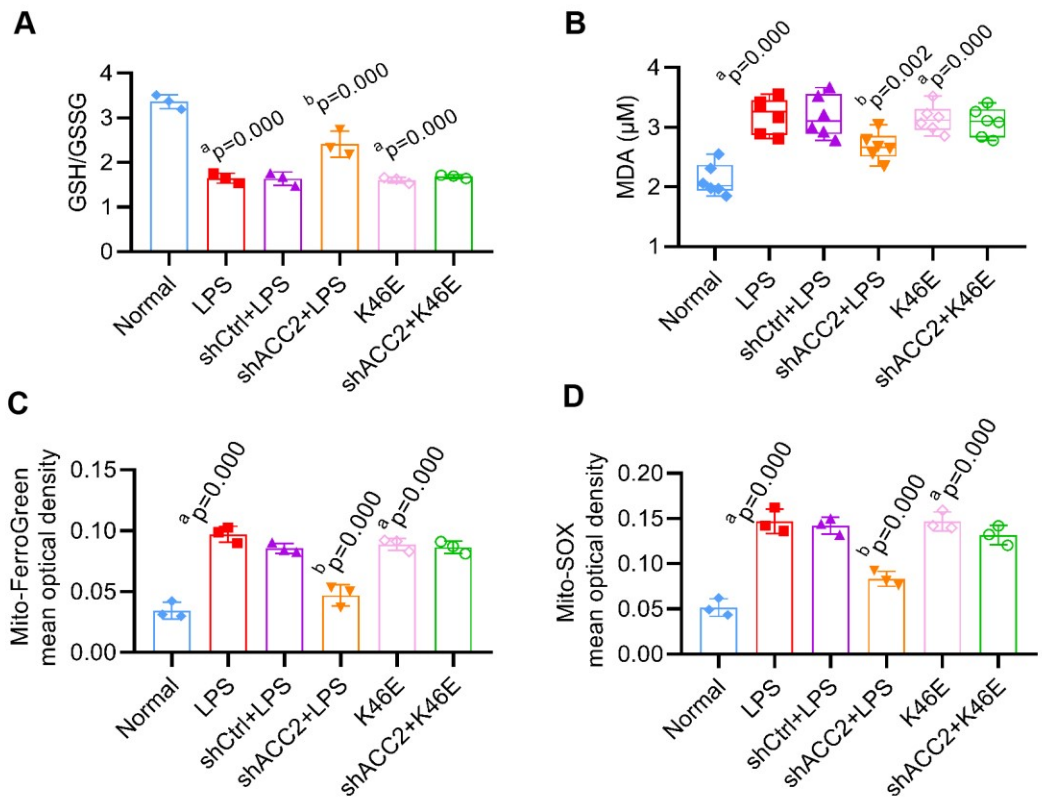

Supplementary Figure 8. The level of **(A)** GSH/GSSG and **(B)** MDA of H9C2 cells after transfected with Ad-Vector and Ad-shACC2 (n=3 independent experiments). (**C**) The mean optical density of Mito-FerroGreen in H9C2 cells (n=3 independent experiments). (**D**) The mean optical density of Mito-SOX in H9C2 cells (n=3 independent experiments). The results were analyzed by one-way ANOVA. a: $p < 0.05$ as compared with the normal group. b: $p < 0.05$ as compared with the LPS group.

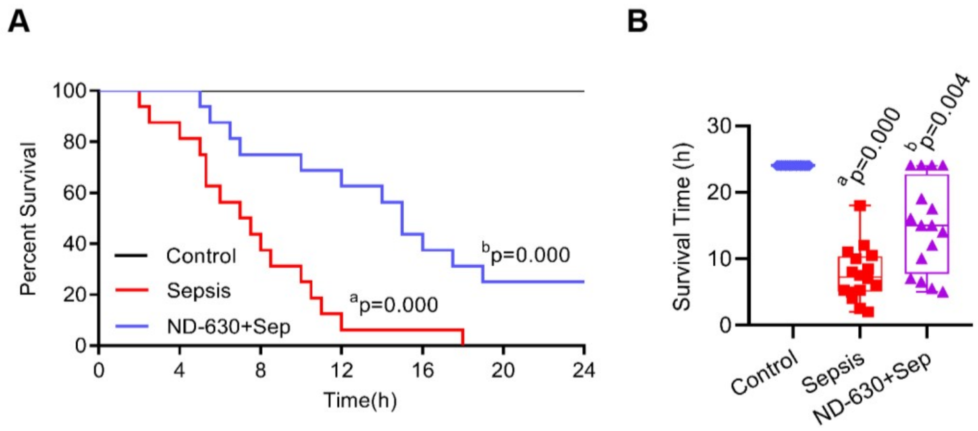

Supplementary Figure 9. (A-B) Survival rate and survival time of sepsis rats after treated with ND-630 (n=16 per group). Rat survival was analyzed by Kaplan-Meier analysis. a: $p < 0.05$ as compared with the control group. b: $p < 0.05$ as compared with the sepsis group.

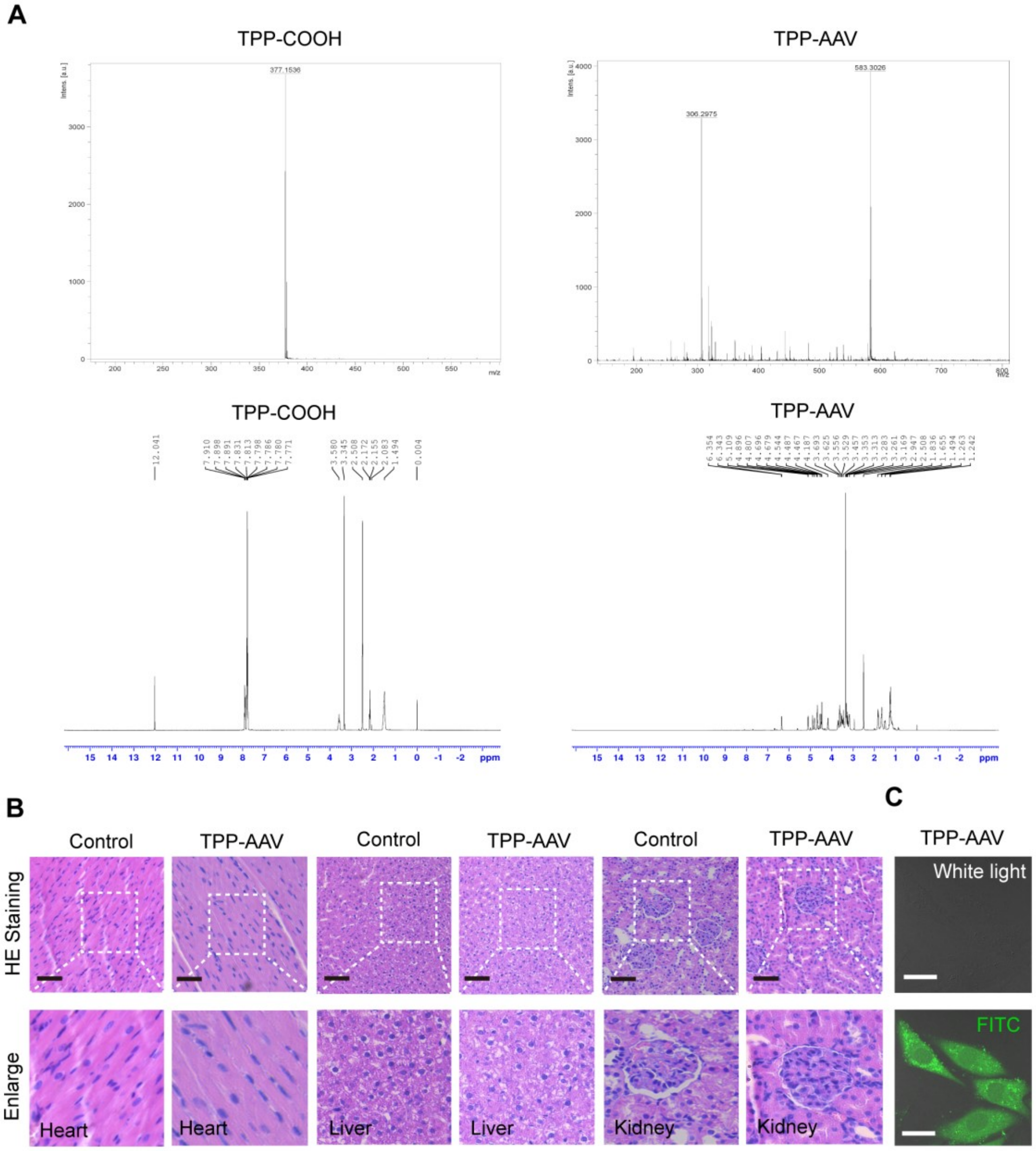

Supplementary Figure 10. (A) The Mass spectrometry and NMR determination of TPP-COOH and TPP-AAV. **(B)** Representative HE staining of rat heart, liver, and kidney (Bar=50μm) (n=6 each group). **(C)** Images of H9C2 cells transfected with TPP-AAV (Bar=25μm) (n=3 independent experiments).
